# Advancing neurosurgery education in junior doctors and medical students – A neurosurgery virtual lecture series

**DOI:** 10.1016/j.amsu.2021.102990

**Published:** 2021-10-30

**Authors:** Maaz A. Khan, Amin Al-Hussainy, Amir Ahmed, Sofyan Al Shdefat

**Affiliations:** University of Cambridge School of Clinical Medicine, Cambridge Biomedical Campus, Box 111, Cambridge, CB2 0SP, UK

**Keywords:** Neurosurgery, Teaching, Medical education, Remote teaching

To the Editor,

As final year medical students at the University of Cambridge, we read with great interest the article titled *“Advancing neurosurgery education in junior doctors and medical students – A neurosurgery virtual lecture series”* by Jing Xian Lee and Ish Ahmed [1]. The authors describe the success of a virtual teaching program at the University Hospital of North Tees. Junior doctors and medical students exhibited an improvement in both their subjective (reported self-confidence) and objective (single best answer [SBA] test scores) measures of knowledge after attending six weeks of lectures once a week on Zoom. We expect this will lead to better care for neurosurgical patients.

We note that foundation doctors reported a greater pre-lecture confidence level than medical students, yet their median pre-lecture SBA scores were not statistically significantly different, except for in Neurointensive Care. We hypothesise that “confidence levels” refer not only to SBA knowledge but also to the ability of the medical student/foundation doctor to manage a patient in that scenario. We surmise that foundation doctors expressed greater confidence in their abilities because of their superior clinical and practical skills, which would support them when managing patients. We therefore highlight the importance of teaching clinical skills in a neurosurgical teaching program. This is challenging to deliver virtually. In a recent study by Abbasi and colleagues assessing e-learning perception and satisfaction in 1225 health sciences students, although 41.3% agreed or strongly agreed that e-learning was good for theoretical knowledge, only 13.4% agreed or strongly agreed that e-learning was good for clinical and practical skills, and only 11.4% felt confident to practice on patients after e-learning [2]. This is consistent with feedback from the present neurosurgery teaching programme, wherein participants suggested simulation would improve the teaching course [1]. However, in sharp contrast to the findings by Abbasi [2], an earlier study by Gormley and colleagues investigated the perception of 269 undergraduate medical students to e-learning, and showed that the vast majority (90.6%) agreed or strongly agreed that, overall, e-learning was useful for learning clinical skills [3]. In particular, the students agreed or strongly agreed that demonstration videos (94%), images (92.6%) and clinical skill checklists (88.4%) were the most useful media to learning clinical skills [3]. We therefore suggest incorporating these into the teaching program and providing this material prior to the start of the lecture. Handouts with high-resolution images will allow subtle features of the images to be appreciated – these are generally lost when screensharing on Zoom due to video compression. We also recommend using the Zoom feature “breakout rooms” to divide students into smaller groups so that they can participate in simulated patient exercises. This will build their confidence and develop their communication skills, which can later be assessed via virtual Objective Structured Clinical Examinations (OSCEs) [4] or virtual structured viva voce examinations (vivas) [5].

Furthermore, the lectures were recorded for those unable to attend live. We are interested in seeing if the degree of improvement was similar in students who attended the Zoom lecture live versus those who only watched the recordings. The sample size might be too small to perform this level of analysis, which is a known limitation of the study. We note that the study only contained one 5th-year medical student. We believe that this was due to the timing of the lecture course, which ran from October to November. The is a notoriously busy period for final year medical students, who have to juggle Foundation Programme Application System (FPAS) applications, medical school finals and the Situational Judgement Test (SJT), alongside clinical placements. Nevertheless, given the clear success of this virtual lecture series, we believe it should continue throughout the year. Moreover, we would like to explore the long-term impact of this teaching program. Re-examining the participants in six months' time will elucidate whether they have retained this knowledge; we hypothesise that participants' knowledge will decline over time. Consequently, organising future revision sessions is crucial. Additional lectures should also be arranged in other areas of neurosurgery that were not covered as standalone topics in the teaching program, such as neuro-oncology. This teaching course could also be extended to other specialties. We believe that these measures will further improve participants’ understanding of clinical medicine and considerably enhance patient care.

## Provenance and peer review

Not commissioned, externally peer reviewed.

## Ethical approval

Ethical approval was not sought.

## Sources of funding

None of the authors received any sources of funding to produce this manuscript.

## Author contribution

All authors conceived the idea, contributed towards the design, and wrote the manuscript. All authors critically reviewed the manuscript and provided final approval. All authors agree to be accountable for all aspects of the work.

## Registration of research studies

1. Name of the registry: N/A
2. Unique Identifying number or registration ID: N/A
3. Hyperlink to your specific registration (must be publicly accessible and will be checked): N/A

## Guarantor

Maaz A. Khan.

## Consent statement

None.

## Declaration of competing interest

None.
